# Supplementary material for: Understanding Type 2 Diabetes Mellitus Risk Parameters through Intermittent Fasting: A Machine Learning Approach
Source: Nutrients. 2023 Sep 10;15(18):3926. doi: 10.3390/nu15183926 (PMC10535779; doi:10.3390/nu15183926)
Supplement: Supplementary file 1 [file nutrients-15-03926-s001.zip › nutrients-2572042-supplementary.pdf]

Table S1 Select the best match of intermittent fasting approach for a prediabetes individual. Input: a prediabetes man, 60 years old, weight 103 kg, BMI 30 and basal fasting glucose of 8.1 mmol/L (145.8 mg/dL)

Intervention	Continues difference Random Forest
**Diet Hi Carb**	0.834
**Diet Hi Mono**	**0.988**
**CER**	0.846
**IER**	0.877
**DMF**	0.874
**FESD**	0.619
**IECR**	0.821
**IECR+PF**	0.711
**IF100**	0.947
**IF70**	0.715
**DR70**	0.858
**CCR**	0.779
**ICR**	0.851

Table S2 Select the best match of intermittent fasting approach for a prediabetes individual. Input: a prediabetes man, 58 years old, weight 106.3 kg, BMI 34.71 and basal fasting glucose of 5.61 mmol/L (101 mg/dL)

Intervention	Continues difference Random Forest
**Diet Hi Carb**	0.217
**Diet Hi Mono**	0.239
**CER**	0.245
**IER**	0.221
**DMF**	0.200
**FESD**	0.048
**IECR**	0.269
**IECR+PF**	0.199
**IF100**	0.292
**IF70**	0.124
**DR70**	0.243
**CCR**	**0.448**
**ICR**	0.190

Table S3: Different cutoff values for difference 15%

Normal values : 5.56 mmol/liter fasting glucose and 80 pmol/liter fasting insulin

Normal value HOMA-IR : 5.56 ***6 * 80 * 18 = 48038

Difference : 0.834 mmol/liter fasting glucose and 12 pmol/liter fasting insulin

HOMA-IR Difference: 7206

Fasting Glucose success : 24 out of 474

Fasting Glucose success, no control : 19 out of 413

HOMA-IR success : 150 out of 471

HOMA-IR success, no control : 126 out of 410

A

		Fasting Glucose				HOMA-IR			
		With control		No control		With control		No control	
Discrete difference above 15%.	J48	0.79	93%	0.82	96%	0.74	74%	0.73	74%
	LMT	0.90	94%	0.91	96%	0.83	75%	0.89	82%
	Random forest	0.90	95%	0.93	96%	0.82	76%	0.87	79%
	Logistic	0.82	95%	0.82	96%	0.83	76%	0.88	82%
Discrete difference above 15%.No interventions	J48	0.64	93%	0.73	94%	0.75	75%	0.74	73%
	LMT	0.91	95%	0.90	95%	0.82	74%	0.82	76%
	Random forest	0.91	95%	0.92	95%	0.82	76%	0.82	75%
	Logistic	0.90	95%	0.94	95%	0.83	77%	0.82	78%

B

		Fasting Glucose RANDOM				HOMA-IR RANDOM			
		With control		No control		With control		No control	
Discrete difference above 15%.	J48	0.45	95%	0.47	95%	0.50	74%	0.50	66%
	LMT	0.50	95%	0.50	95%	0.49	76%	0.49	70%
	Random forest	0.65	95%	0.56	95%	0.50	75%	0.49	69%
	Logistic	0.56	95%	0.57	95%	0.48	76%	0.49	70%
Discrete difference above 15%.No interventions	J48	0.45	93%	0.47	95%	0.50	76%	0.50	70%
	LMT	0.50	95%	0.50	95%	0.50	76%	0.50	71%
	Random forest	0.50	95%	0.60	95%	0.51	73%	0.53	68%
	Logistic	0.62	95%	0.68	95%	0.50	76%	0.51	70%

Table S4: Different cutoff values for difference 10%

Normal values : 5.56 mmol/liter fasting glucose and 80 pmol/liter fasting insulin

Normal value HOMA-IR : 5.56 *6 * 80 * 18 = 48038

Difference : 0.556 mmol/liter fasting glucose and 8 pmol/liter fasting insulin

HOMA-IR Difference: 4803

Fasting Glucose success : 61 out of 474

Fasting Glucose success, no control : 52 out of 413

HOMA-IR success : 205 out of 471

HOMA-IR success, no control : 175 out of 410

A

		Fasting Glucose		HOMA-IR	
		With control	No control	With control	No control
Discrete difference above 10%.	J48	0.67 85%	0.66 86%	0.70 69%	0.79 75%
	LMT	0.77 87%	0.75 88%	0.80 74%	0.89 79%
	Random forest	0.74 87%	0.76 88%	0.80 72%	0.87 79%
	Logistic	0.74 88%	0.75 88%	0.80 75%	0.88 78%
Discrete difference above 10%.No interventions	J48	0.49 87%	0.55 88%	0.71 71%	0.77 75%
	LMT	0.76 87%	0.76 87%	0.77 71%	0.84 76%
	Random forest	0.72 87%	0.73 87%	0.77 73%	0.87 79%
	Logistic	0.79 87%	0.78 86%	0.80 73%	0.88 78%

B

		Fasting Glucose RANDOM		HOMA-IR RANDOM	
		With control	No control	With control	No control
Discrete difference above 10%.	J48	0.49 87%	0.48 87%	0.50 52%	0.56 56%
	LMT	0.59 87%	0.50 87%	0.60 56%	0.57 54%
	Random forest	0.56 87%	0.57 87%	0.58 57%	0.59 57%
	Logistic	0.58 87%	0.48 87%	0.60 57%	0.58 55%
Discrete difference above 10%.No interventions	J48	0.49 87%	0.48 87%	0.51 53%	0.58 57%
	LMT	0.60 87%	0.50 87%	0.60 59%	0.55 55%
	Random forest	0.53 84%	0.56 86%	0.55 56%	0.57 54%
	Logistic	0.57 87%	0.57 87%	0.60 59%	0.60 57%

Table S5: Different cutoff values for difference 20%

Normal values : 5.56 mmol/liter fasting glucose and 80 pmol/liter fasting insulin

Normal value HOMA-IR : 5.56 *6 * 80 * 18 = 48038

Difference : 1.112 mmol/liter fasting glucose and 16 pmol/liter fasting insulin

HOMA-IR Difference: 9608

Fasting Glucose success : 10 out of 474

Fasting Glucose success, no control : 8 out of 413

HOMA-IR success : 119 out of 471

HOMA-IR success, no control : 99 out of 410

		Fasting Glucose				HOMA-IR			
		With control		No control		With control		No control	
Discrete difference above 20%.	J48	0.48	98%	0.56	98%	0.77	79%	0.76	77%
	LMT	0.92	98%	0.90	99%	0.85	81%	0.85	81%
	Random forest	0.85	98%	0.83	98%	0.86	81%	0.86	81%
	Logistic	0.74	88%	0.80	98%	0.84	81%	0.86	81%
Discrete difference above 20%.No interventions	J48	0.54	98%	0.40	98%	0.80	79%	0.77	79%
	LMT	0.88	98%	0.87	99%	0.85	81%	0.86	82%
	Random forest	0.70	98%	0.74	98%	0.86	79%	0.87	80%
	Logistic	0.87	98%	0.91	98%	0.86	81%	0.86	82%

Table S6: Random test for continuous difference

Fasting Glucose total : 474

Fasting Glucose total, no control : 413

HOMA-IR total : 471

HOMA-IR total, no control : 410

		Fasting Glucose		HOMA-IR	
		With control	No control	With control	No control
Continuous difference	Random Forest	0.51	0.51	0.36	0.46
Continuous difference random	Random Forest	-0.13	-0.088	0.017	-0.083

Table S7: Area Under Curve (AUC) and Accuracy of predicting fasting glucose or HOMA-IR difference.

Each cell in the table contains two lines. The upper line shows the AUC results while the lower line describes the accuracy results. Every line composed of 3 columns the left column shows the result of 10-fold test, the middle column shows the results of splitting 20% of the data as test dataset test while the right column (in parentheses) contains the training results.

		Fasting Glucose		HOMA-IR	
		With control	No control	With control	No control
Discrete difference	J48	0.66 0.61 (0.81)<br>65% 62% (69%)	0.67 0.78 (0.83)<br>67% 67% (69%)	0.68 0.6 (0.87)<br>70% 68% (85%)	0.65 0.7 (0.85)<br>68% 65% (84%)
	LMT	0.72 0.73 (0.77)<br>67% 64% (69%)	0.73 0.74 (0.78)<br>66% 64% (68%)	0.60 0.6 (0.62)<br>72% 69% (85%)	0.70 0.7 (0.72)<br>73% 77% (78%)
	Random forest	0.71 0.71 (0.82)<br>68% 66% (73%)	0.70 0.74 (0.76)<br>65% 67% (69%)	0.68 0.66 (0.85)<br>70% 68% (93%)	0.71 0.75 (0.87)<br>71% 70% (93%)
	Logistic	0.72 0.81 (0.88)<br>68% 66% (71%)	0.73 0.88 (0.88)<br>66% 69% (69%)	0.70 0.62 (0.74)<br>71% 67% (73%)	0.70 0.73 (0.74)<br>74% 76% (76%)
Discrete difference. No interventions	J48	0.61 0.60 (0.61)<br>63% 62% (66%)	0.63 0.82 (0.85)<br>65% 66% (66%)	0.57 0.58 (0.69)<br>68% 62% (76%)	0.54 0.61 (0.64)<br>68% 71% (78%)
	LMT	0.70 0.71 (0.72)<br>64% 62% (65%)	0.72 0.75 (0.77)<br>66% 65% (67%)	0.65 0.6 (0.73)<br>70% 69% (80%)	0.62 0.62 (0.64)<br>70% 74% (74%)
	Random forest	0.68 0.69 (0.77)<br>63% 62% (68%)	0.68 0.74 (0.76)<br>64% 64% (69%)	0.60 0.61 (0.78)<br>69% 65% (93%)	0.63 0.65 (0.8)<br>72% 69% (96%)
	Logistic	0.71 0.72 (0.74)<br>65% 61% (65%)	0.71 0.71 (0.72)<br>66% 65% (67%)	0.65 0.59 (0.66)<br>70% 66% (71%)	0.64 0.66 (0.78)<br>71% 75% (75%)
Discrete difference above 15%.	J48	0.79 0.64 (0.96)<br>93% 91% (98%)	0.82 0.92 (0.97)<br>96% 96% (98%)	0.74 0.65 (0.93)<br>74% 72% (89%)	0.73 0.81 (0.96)<br>74% 73% (92%)
	LMT	0.90 0.91 (0.94)<br>94% 92% (97%)	0.91 0.93 (0.97)<br>96% 94% (98%)	0.83 0.69 (0.74)<br>75% 72% (88%)	0.89 0.82 (0.82)<br>82% 81% (81%)
	Random forest	0.90 0.89 (1.0)<br>95% 93% (100%)	0.93 0.94 (0.98)<br>96% 96% (98%)	0.82 0.81 (1.0)<br>76% 75% (100%)	0.87 0.88 (1.0)<br>79% 76% (100%)
	Logistic	0.82 0.90 (0.96)<br>95% 92% (97%)	0.82 0.97 (0.97)<br>96% 97%(97%)	0.83 0.75 (0.87)<br>76% 72% (78%)	0.88 0.91 (0.92)<br>82% 84% (84%)
Discrete difference above 15%.No interventions	J48	0.64 0.61 (0.93)<br>93% 94% (96%)	0.73 0.92 (0.92)<br>94% 95%(95%)	0.75 0.7 (0.87)<br>75% 69% (83%)	0.74 0.8 (0.85)<br>73% 76% (83%)
	LMT	0.91 0.92 (0.93)<br>95% 92% (95%)	0.90 0.93 (0.95)<br>95% 94% (96%)	0.82 0.7 (0.9)<br>74% 74% (85%)	0.82 0.82 (0.84)<br>76% 81% (81%)
	Random forest	0.91 0.92 (1.0)<br>95% 94% (100%)	0.92 0.98 (1.0)<br>95% 95%(100%)	0.82 0.77 (1.0)<br>76% 72% (100%)	0.82 0.85 (1.0)<br>75% 73% (100%)
	Logistic	0.90 0.91 (0.93)<br>95% 91% (95%)	0.94 0.94 (0.95)<br>95% 94% (96%)	0.83 0.72 (0.84)<br>77% 73% (78%)	0.82 0.84 (0.86)<br>78% 82% (82%)
Continuous difference	Random Forest	0.51	0.51	0.36	0.46
